# iSignDB: A database for smartphone signature biometrics

**DOI:** 10.1016/j.dib.2020.106597

**Published:** 2020-11-28

**Authors:** Suraiya Jabin, Sumaiya Ahmad, Sarthak Mishra, Farhana Javed Zareen

**Affiliations:** Department of Computer Science, Faculty of Natural Sciences, Jamia Millia Islamia, New Delhi 110025, India

**Keywords:** Signature, Verification, Authentication, Sensor, Smartphone, Biometric signature, Behavioural analysis

## Abstract

The signature has long been in use for the user verification. These signatures have user specific features that differentiate the individual for authentication. The signature verification can be offline or online. The offline verification considers only the static features of the signatures through the signature image, while the online verification considers various dynamic features associated with the signature such as pen pressure, pen tilt angle, velocity, acceleration, pen up and pen down, etc at various time stamps which are recorded using special digitizing tablets such as Wacom devices (STU-500, STU-530 and DTU-1031) [1,14] etc. In todays scenario, smartphones are widely used world-wide, and come equipped with various sensors e.g. accelerometer, gyroscope, magnetometer, GPS, etc. able to capture sensor logs and have been used widely in the literature to capture the dynamics of users’ behaviour while a signer signs on his smartphone. However, there is scarcity of publicly available databases for the online signatures collected using smartphone. In the present work, we describe biometric signature dataset iSignDB captured using smartphone.

The iSignDB [6,10] consists of the genuine signature samples of a user as well as the skilled forgery samples where imposter was given multiple attempts to mimic the mannerisms of the original signer before giving skilled forgery samples. A total of 30 samples towards the genuine signature over 3 sessions with 10 samples per session while 15 samples of the skilled forgery with 5 samples per session were collected. Each of the session were at least 15 days apart. The iOS and Android based smartphones (namely iPhone7 and Redmi Note 7) were used for the data collection.

The sensors used to collect this data, present in the smartphone are the gyroscope, magnetometer, GPS, and accelerometer. Smartphones having sensors any one lesser than these four, were not used for data collection, in order to have consistent number of features under each sample. They generate the following sensor readings: angular velocity, acceleration, orientation, geomagnetic field in the x, y, and z directions, position, which is collected using the MATLAB Mobile App installed in the smartphone, that sends the data to a licensed MathWorks cloud account in the form of a multitude of sensor logs. Each sample has image of the signature along with sensor readings.

Some of the publicly available smartphone biometric signature databases are DooDB [2], MOBISIG [3], eBioSign DS 2 [7], etc. in which at least acceleration sensor reading is present but the iSignDB ensures these five of the sensor readings (acceleration, angular velocity, magnetic field, orientation, position) under each sample. This dataset can be successfully used to design smartphone biometric signature authentication system which is robust against a number of spoof attacks [11], [12], [13], [14]. As every user has a unique way of handling his/her smartphone which varies over different level of emotional intelligence of the user over a time period, this dataset can also be used for behavioural analysis of the users.

## Specifications Table

**Table:** 

Subject	Data Science Applied Machine Learning
Specific subject area	-This dataset can be used to build biometric signature authentication system using machine learning techniques. The smartphone sensor data is collected while the user signs on the smartphone screen. -It can be used to build a behavioural analysis system as the sensor data is acquired from emotionally intelligent subjects. -Recently, researchers [4,5] have used smartphone sensor data for human activity recognition. As iSignDB has been collected while a user signs on smartphone screen, it can also be deployed to design a human activity recognizer.
Type of data	Image (PNG) and Microsoft Office Excel Comma Separated Values File (.csv) files
How data were acquired	-MATLAB Mobile App installed on (iOS and Android based) smartphones, namely, iPhone 7 and Redmi Note 7 -A Signature capture app just to save image of sign scribbled by finger on a canvas or smartphone screen -A purchased/licensed MATLAB 2020b software
Data format	Raw
Parameters for data collection	• All the volunteers were 22-28 years of age, pursuing master in computer application from a top ranked university (www.jmi.ac.in) in New Delhi, India during 2017-2020. Now most of them are placed in software industries of India with very good packages. • Volunteers were separated into two groups where one group contributed samples towards genuine and another skilled forgery. • Volunteers used smartphones strictly with all 4 sensors present. • Volunteers were explained the process to capture the sensor data contributed under observation.
Description of data collection	The MATLAB Mobile Application installed on the smartphones gets connected to licensed MathWorks cloud account, in order to collect the sensor data of the device while the user signs on the screen of smartphone. The data is collected as follows: Press the start button for sensor data collection on MATLAB Mobile app; sign on canvas with the Signature app; press the stop button on MATLAB Mobile app; save the sensor data and image of signature.
Data source location	Institution: Department of Computer Science, Jamia Millia Islamia City: New Delhi Country: India Latitude and longitude (and GPS coordinates) for collected samples/data: 28.5610° N, 77.2845° E
Data accessibility	-With article in the form of Supplementary file. -Also available at this link: https://github.com/suraiyajabin/iSignDB2020

## Value of the Data

• The hand-written signatures are used for signing cheques, official documents, authentication process etc., almost everywhere. Here we present biometric signature data which is basically a sensor log collected using smartphone while a signer signs on the screen of smartphone. This sensor log is collection of acceleration, angular velocity, magnetic field, orientation, and position of smartphone at various timestamps. This dataset iSignDB [6,10] promotes authentication on the go while remotely accessing banking/official applications.
• This dataset can be used by behavioural scientists, biometrics researchers, psychologists, etc.
• This dataset can be used for building biometric signature authentication system that can be deployed/integrated with internet banking apps being accessed with smartphone to possibly authenticate/verify users, eliminating the use of PINs. Alternatively, biometric signature authentication can be used as another step with passwords/PINs to further strengthen the authentication process.
• This dataset can also be used for doing behavioural/psychological analysis of these 32 users. We collected the samples 15 days apart, so that emotional intelligence of users can be captured.

## 1 Data Description

The database consists of genuine and skilled forgery samples for the biometric signatures of 32 users in the respective directory named as 'genuine' and 'skilled forgery'.

There are 30 genuine samples for each user with 10 samples taken in each of the 3 sessions.

While there are 15 skilled forgery samples for each of the 32 users.

We followed a nomenclature to name various data files which are part of iSignDB. The sensor data is stored as a Microsoft Office Excel Comma Separated Values File (.csv) files. Each of the data file is named as:

u0XX_sY_A0Z_B

where

XX: represents the user number

Y: is the session number

A: can have values "g" and "f" for "genuine" and "skilled forgery" respectively.

Z: is the sample number in the session “sY”.

B: specifies the type of data that can take values

  "Acc" for "Acceleration"

  "AngVel" for "Angular Velocity"

  "MagField" for "Magnetic Field"

  "Orient" for "Orientation"

  "Pos" for "Position"

  "Im" for "image"

The image is the signature image in the '.png' format and the rest are the sensor log files in the format '.csv'.

Both of these data are in the respective directories namely, "Images" and "Sensor Data".

**Fig. 1:**
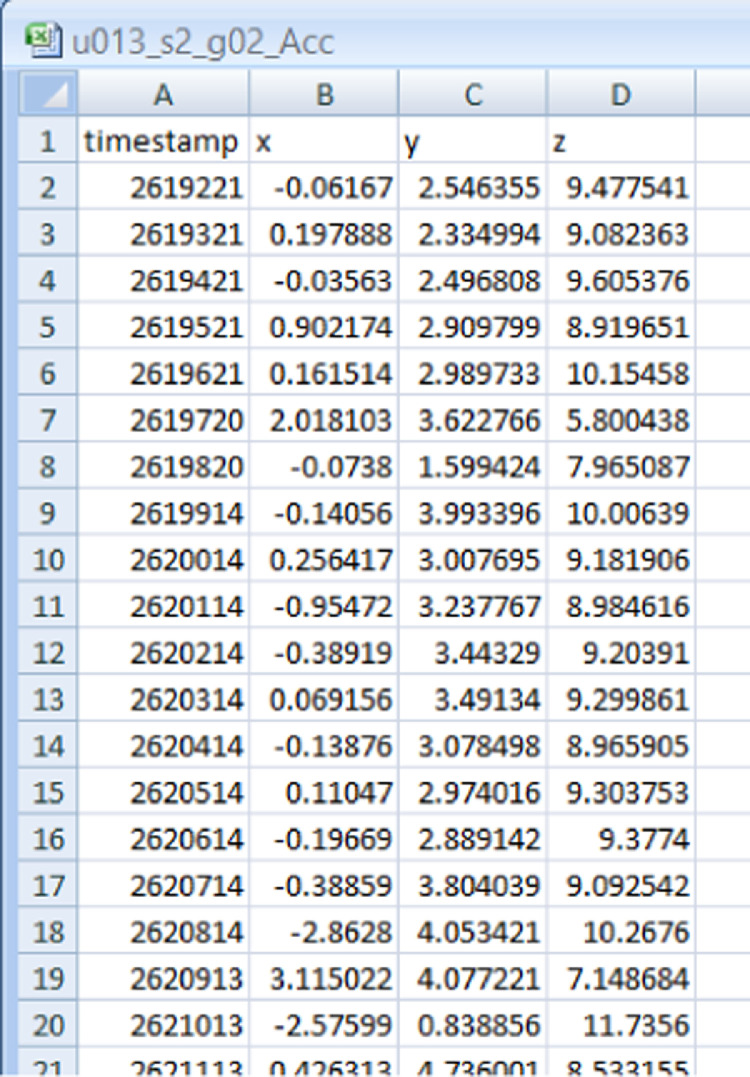
Snapshot of the second sample of genuine -Acceleration(sensor) data of user 13 collected in session 2.

Each of the sensor data file for Acceleration, Angular Velocity, Magnetic Field, Orientation contains 4 columns, the description and headers of each is given below and shown in Fig. 1.

**Table:** 

A	timestamp	specifies the timestamp
B	x	sensor data in the x-direction
C	y	sensor data in the y-direction
D	z	sensor data in the z-direction

**Fig. 2:**
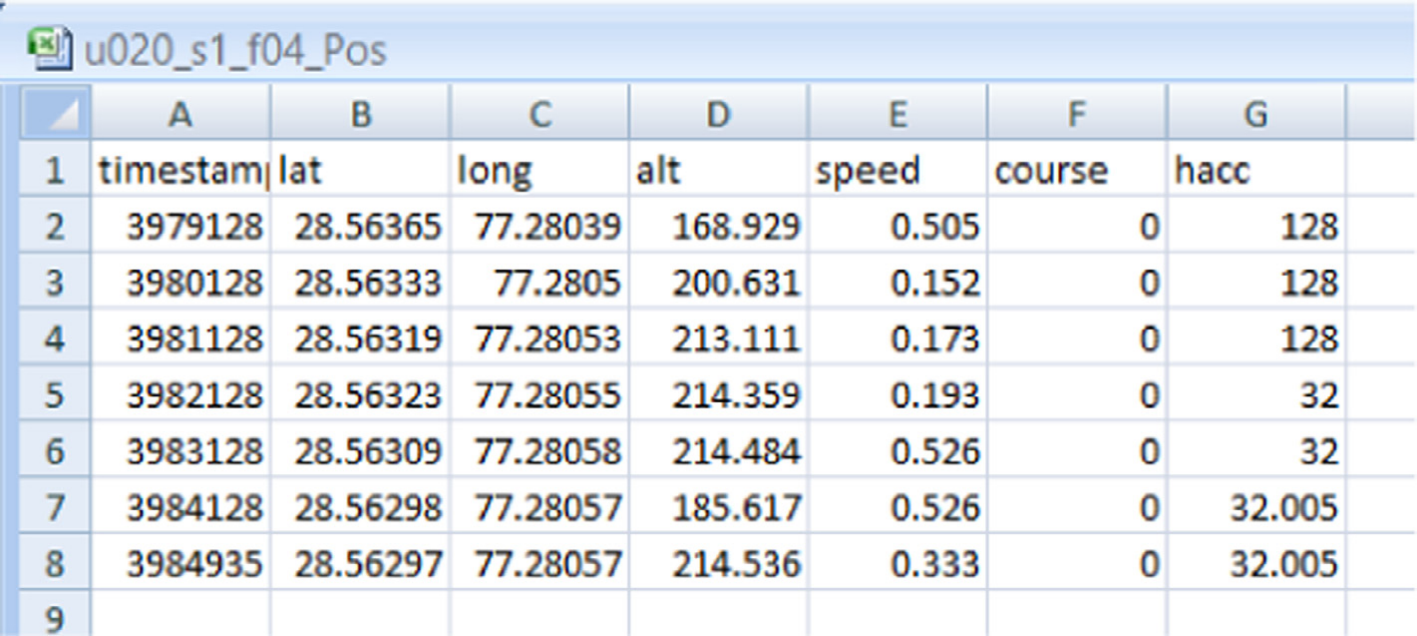
Snapshot of the fourth sample of skilled forgery-Position(sensor) data of user 20 collected in session 1.

The position sensor data files have seven columns, the description and headers of each is shown in Fig. 2 and given below.

**Table:** 

A	timestamp	specifies the timestamp
B	latitude	latitude relative to the equator (degrees)
C	longitude	longitude relative to the zero meridian(degrees)
D	altitude	altitude above sea level (meters)
E	speed	speed (meter/second)
F	course	course relative to true north (degrees)
G	hacc	horizontal accuracy(meters)

The header to each of the sensor data file is also specified as the first row.

As the dataset is collected from a variety of smartphone devices (iOS and Android), each using different foreground and background settings for recording images of signature, images are recorded mainly in two categories:

(i) ‘white sign over black background’ whose preview appears to be white signature over white background.
(ii) ‘black sign over white background’.

The first category is the most suitable format for any kind of processing, and it would not require any preprocessing but the second category will need one level of preprocessing i.e. by taking complement of the image. It would be preferable to open signature images using image processing commands in Python/MATLAB. We have shown how to visualize a signature image in MATLAB using the imread() and imshow() commands.

>> img=imread(' u01_s2_g03_Im.png');

>> imshow(img)

**Fig. 3:**
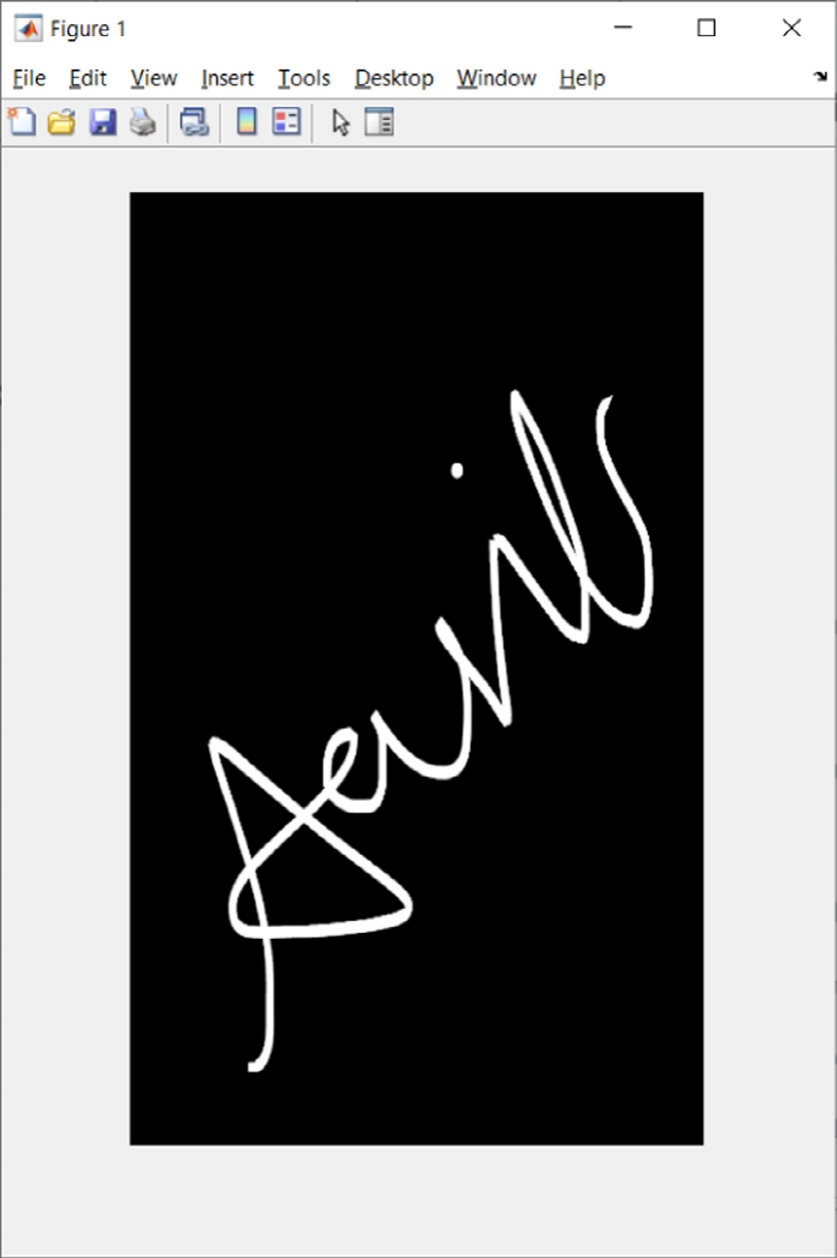
A Signature image opened in MATLAB.

Fig. 3 depicts the signature image in MATLAB as outcome of above two commands.

As this dataset contains biometrics of 32 users, we allow this data set to be available for reuse only after signing a “Term of Use” which is available as a Supplementary file.

## 2 Experimental Design, Materials and Methods

The devices used for data collection are the iOS and Android based smartphones (e.g. iPhone7 and Redmi Note 7) devices for whom all of four sensor readings were available. Each of the device has the MATLAB Mobile App installed for the sensor data collection, and a Signature capture app with a canvas to scribble signature using a finger [8,9]. A licensed MathWorks account is needed with which MATLAB Mobile app can connect to send the sensor data. We used MATLAB 2020b licensed software for the same.

The volunteers were between the age group 22-28 years. Each of the volunteer was explained the process of the data capture which is described below. The data was captured in office-like scenario and fully supervised, thus the samples were re-taken if any mistake was done.

The user connects MATLAB Mobile app to a licensed MathWorks account and then follows the steps given below.

1. The volunteer pressed the 'Start button' on the MATLAB Mobile App to start sending the sensor data to MathWorks cloud
2. The volunteer then signed on the canvas.
3. 'Stop button' was pressed to stop the sensor data sending, after the completion of signature.
4. The data collected was saved.
5. Similarly, steps 1-4 were repeated for each of the signatures.
6. Meanwhile, another volunteer practiced and imitated the signature for the skilled forgery.
7. Similarly, the volunteer who performed the skilled forgery repeated the steps 1-4.

The data was saved on the licensed MathWorks cloud account.

## Ethics Statement

All the users were informed about the data collection process and the data distribution among other researchers that can take place in the future. They willingly contributed to the data collected.

a) The participant data are fully anonymized.
b) The compliance to data redistribution policies from the platform(s).
c) An informed consent of all participants has been obtained.

## Declaration of Competing Interest

The authors declare that they have no known competing financial interests or personal relationships which have or could be perceived to have influenced the work reported in this article.

## Appendix Supplementary materials

Image, application 1

Image, application 2

Image, application 3
