# Supplementary figures and images for: iSignDB: A database for smartphone signature biometrics (part 2 of 2)
Source: Data Brief. 2020 Nov 28;33:106597. doi: 10.1016/j.dib.2020.106597 (PMC7725742; doi:10.1016/j.dib.2020.106597)

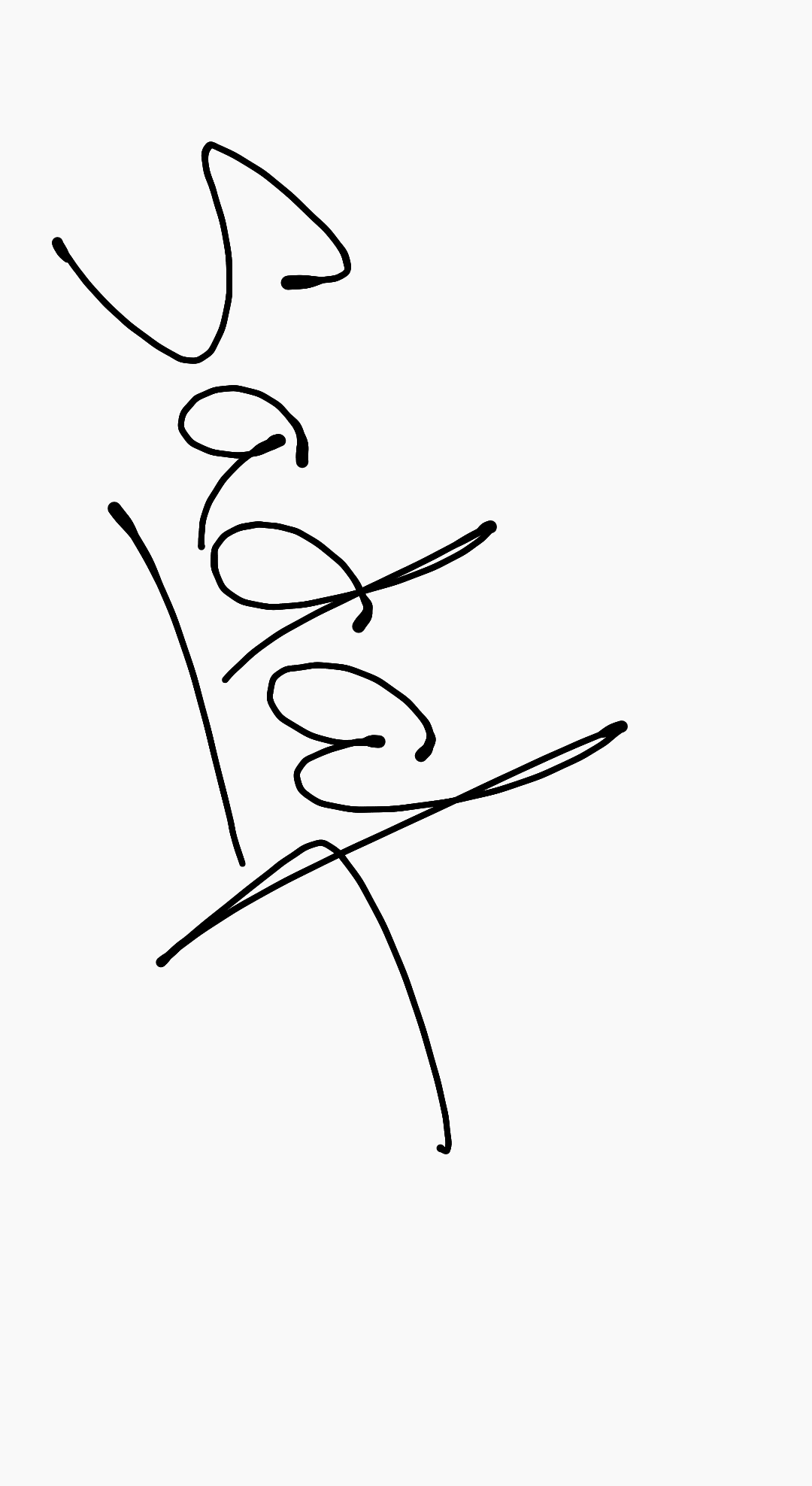

Supplement: Supplementary file 3 [file mmc3.zip › u022/genuine/Images/u022_s3_g03_Im.png]

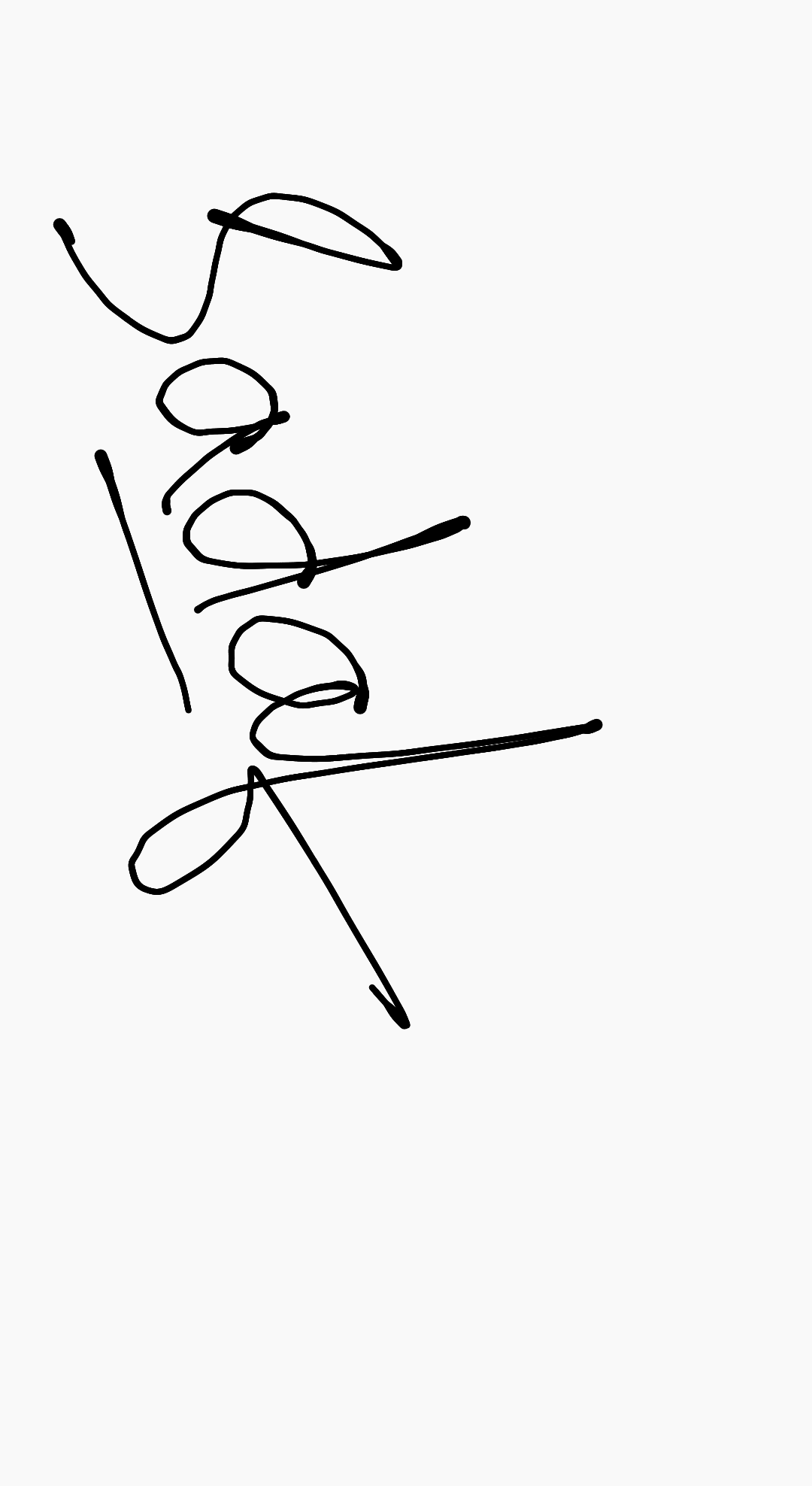

Supplement: Supplementary file 3 [file mmc3.zip › u022/genuine/Images/u022_s3_g04_Im.png]

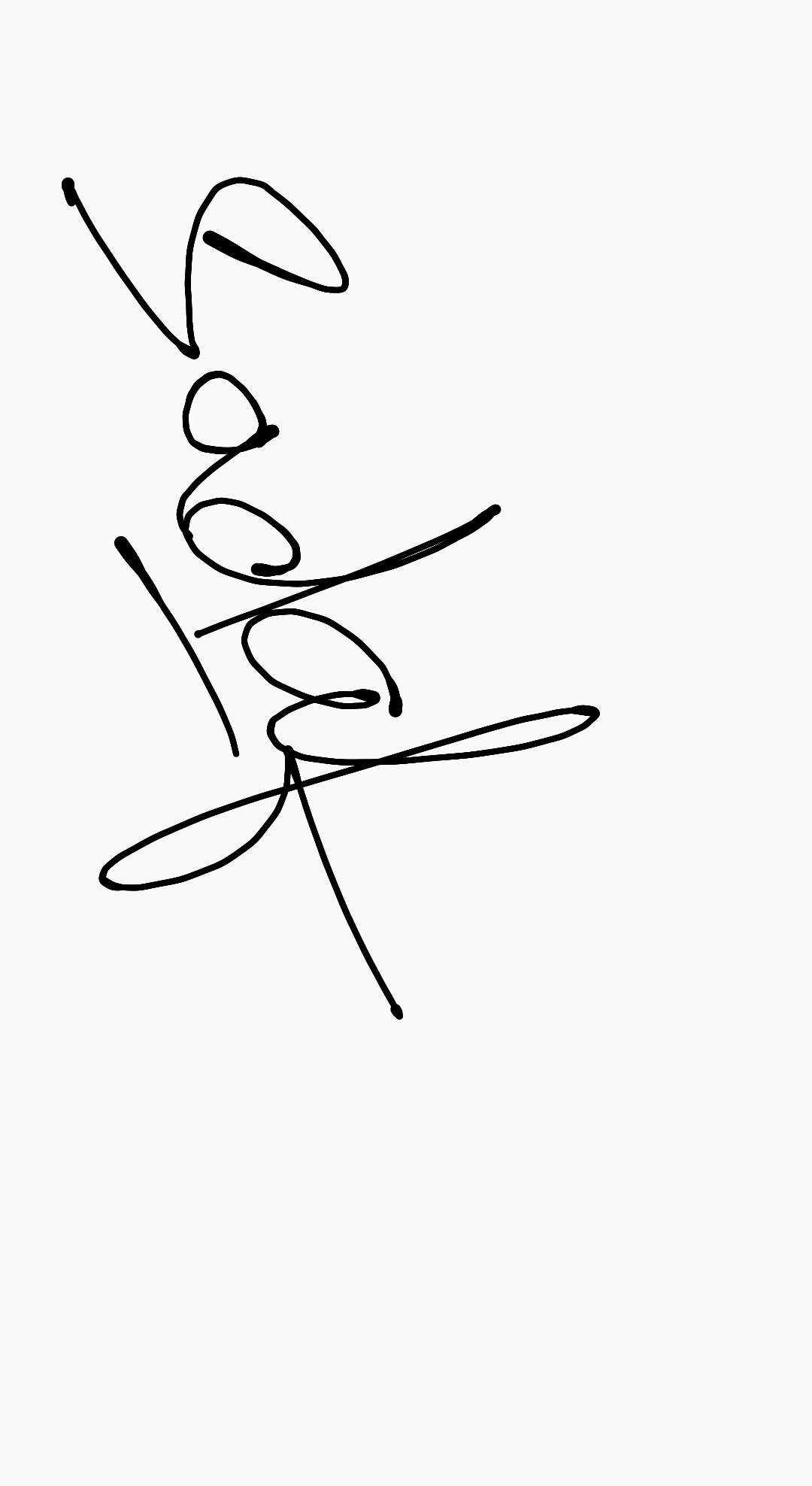

Supplement: Supplementary file 3 [file mmc3.zip › u022/genuine/Images/u022_s3_g05_Im.png]

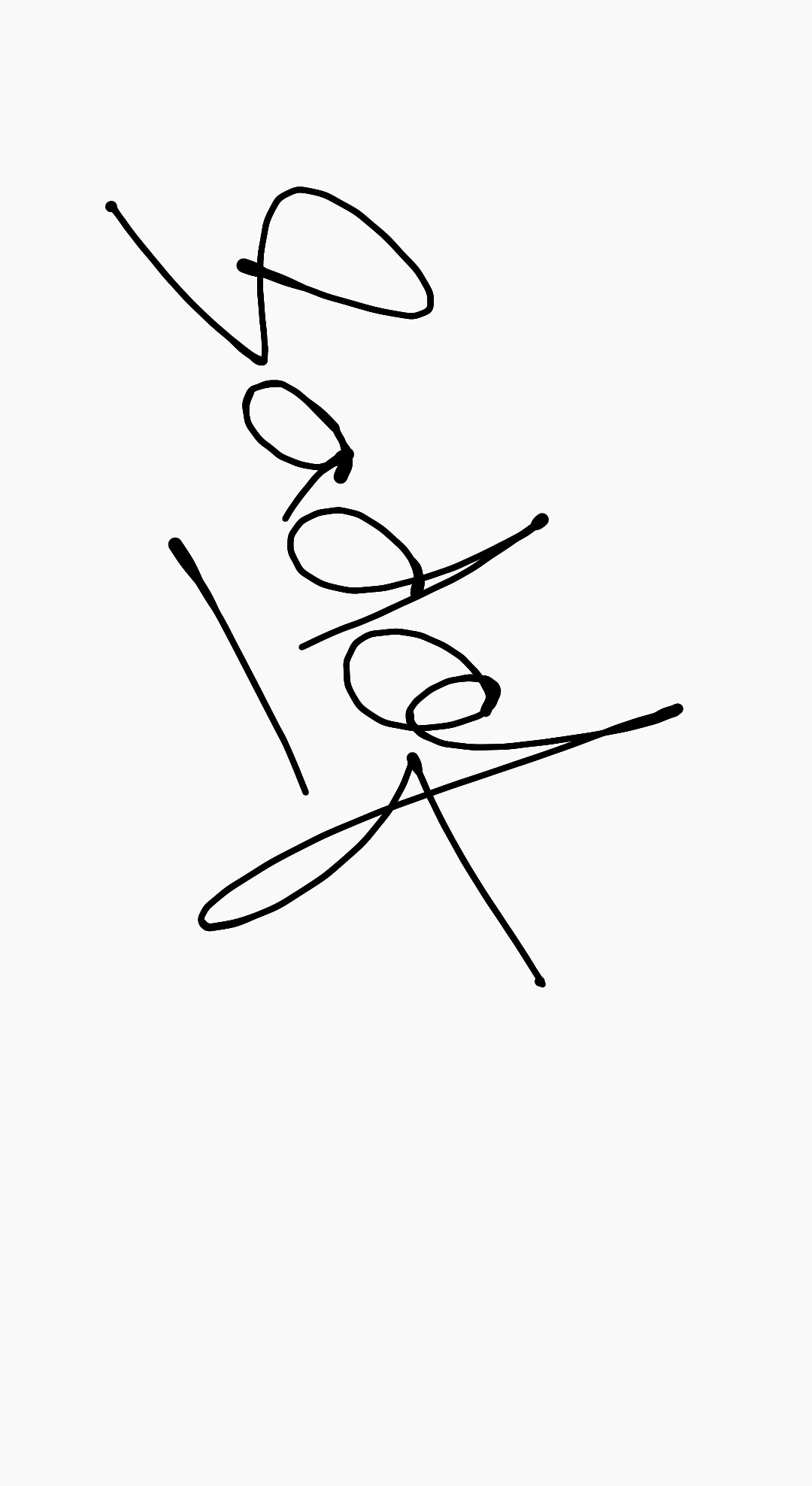

Supplement: Supplementary file 3 [file mmc3.zip › u022/genuine/Images/u022_s3_g06_Im.png]

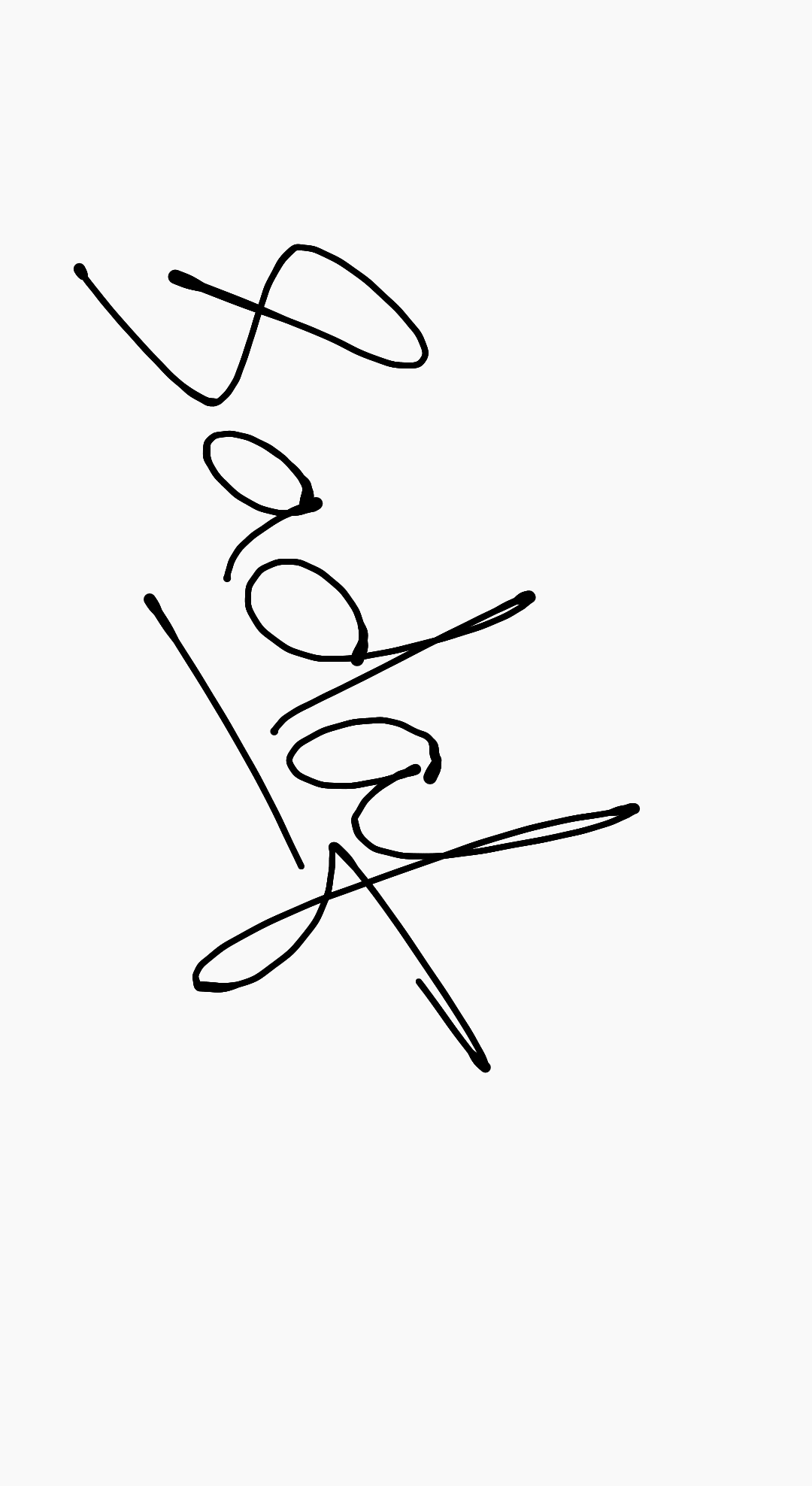

Supplement: Supplementary file 3 [file mmc3.zip › u022/genuine/Images/u022_s3_g07_Im.png]

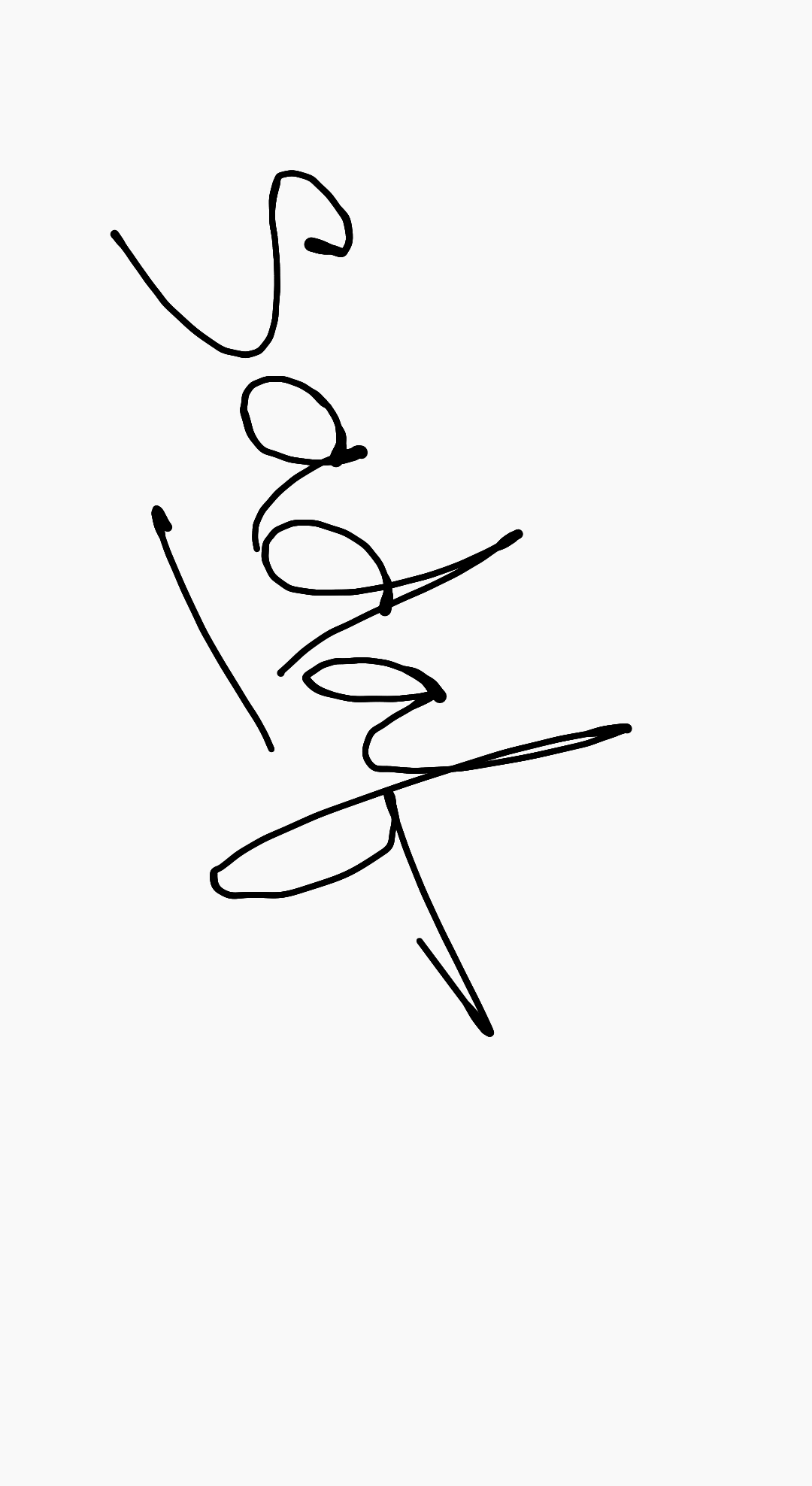

Supplement: Supplementary file 3 [file mmc3.zip › u022/genuine/Images/u022_s3_g08_Im.png]

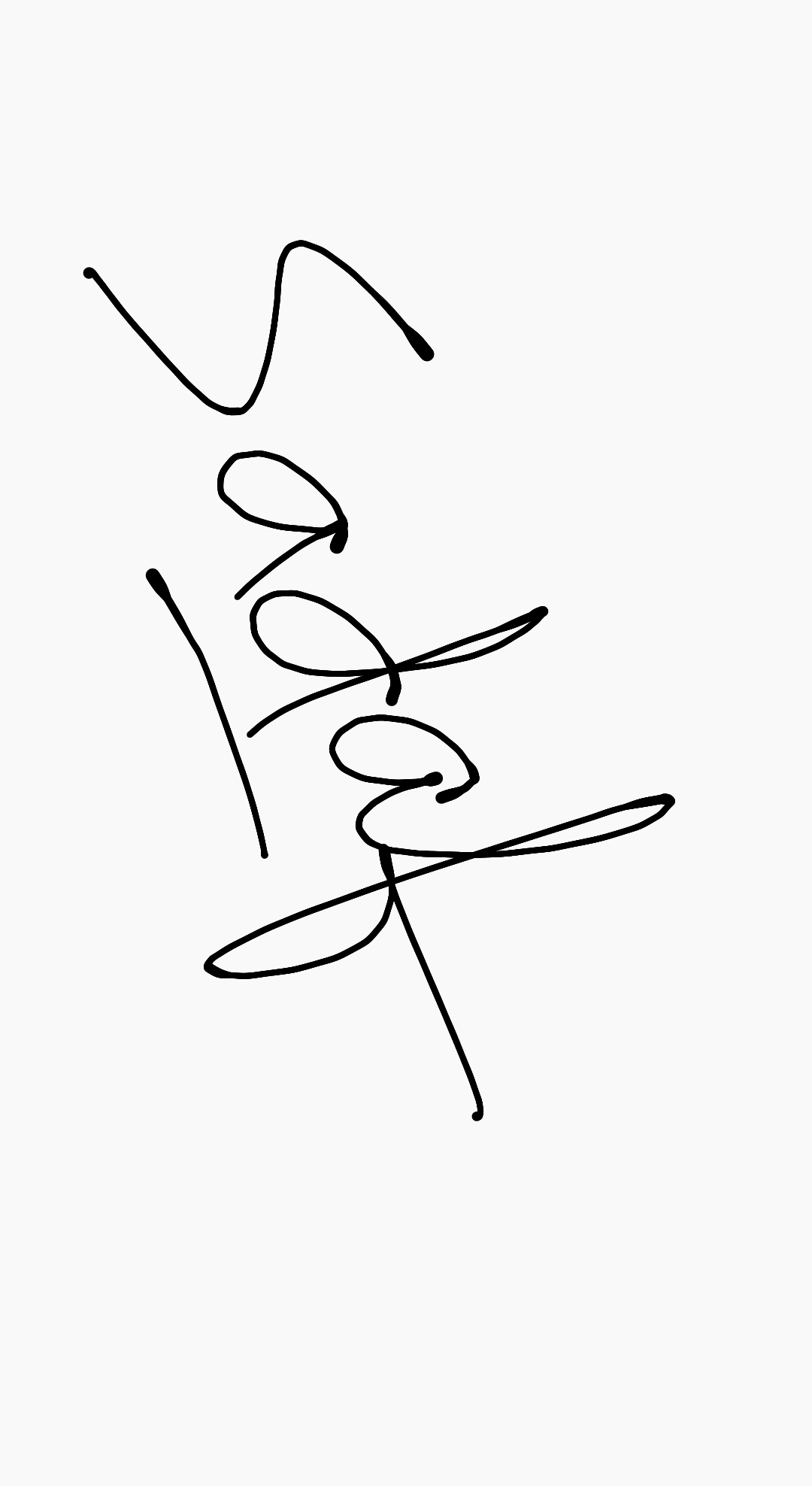

Supplement: Supplementary file 3 [file mmc3.zip › u022/genuine/Images/u022_s3_g09_Im.png]

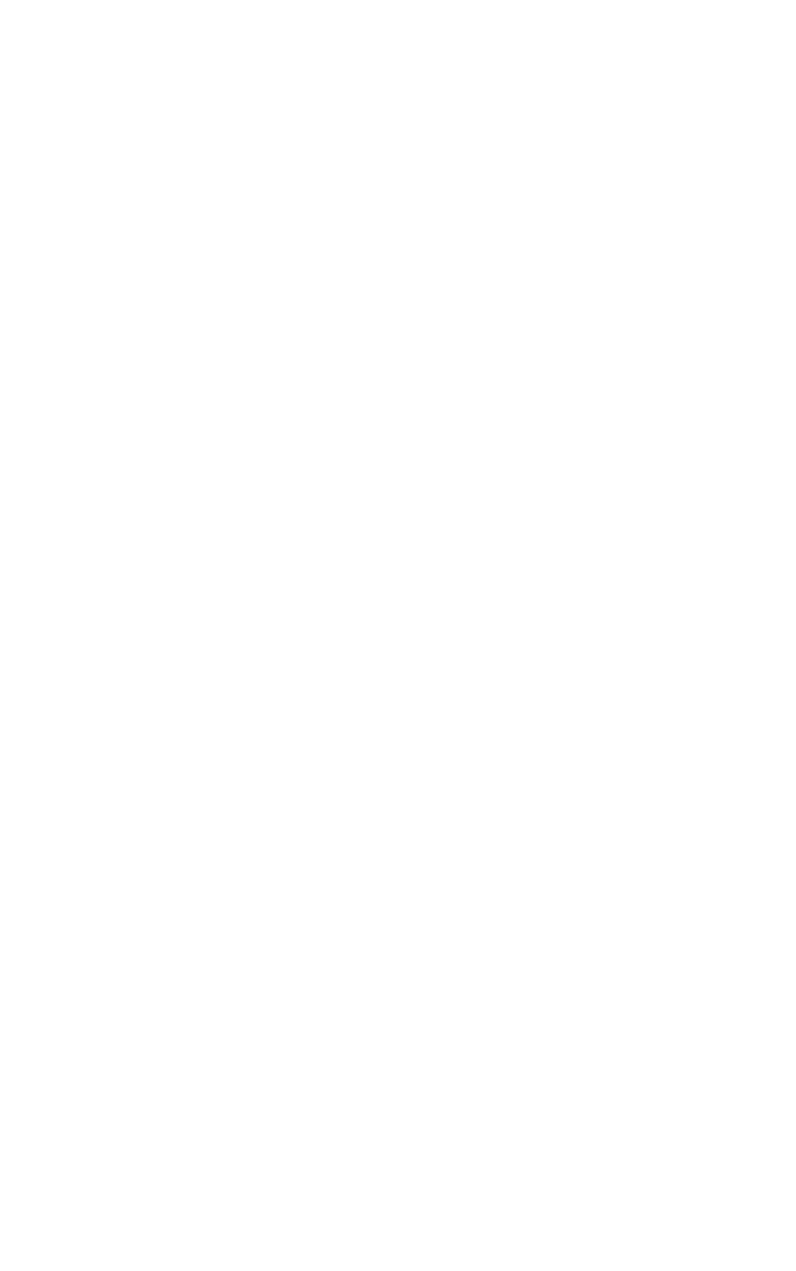

Supplement: Supplementary file 3 [file mmc3.zip › u022/skilled forgery/Images/u022_s1_f01_Im.png]

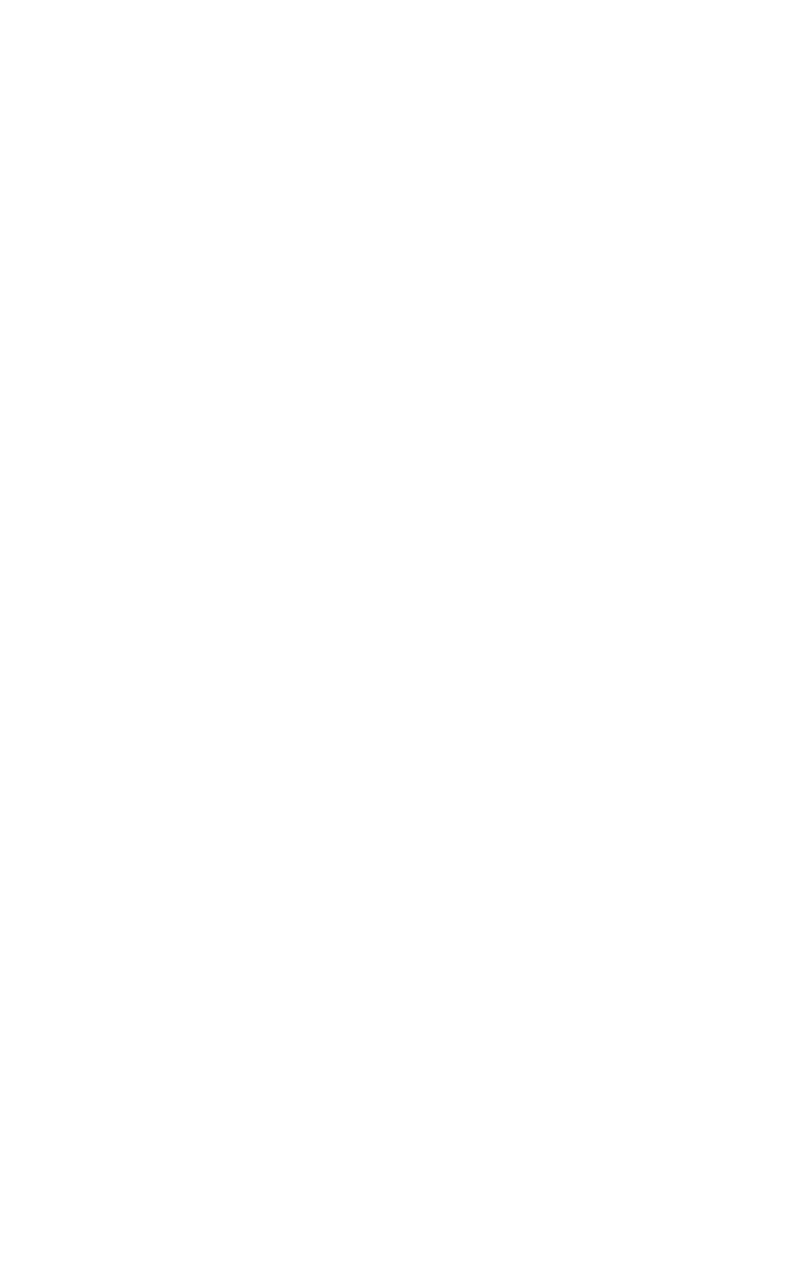

Supplement: Supplementary file 3 [file mmc3.zip › u022/skilled forgery/Images/u022_s1_f02_Im.png]

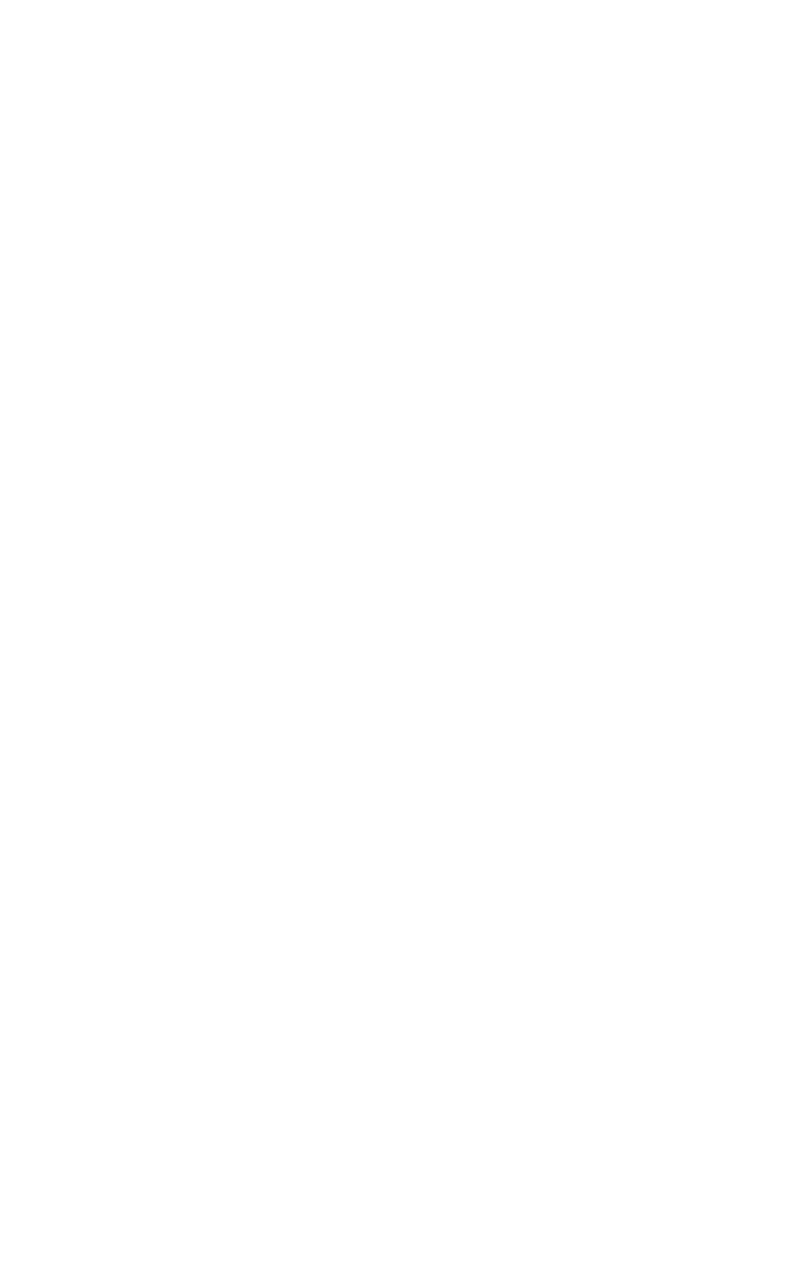

Supplement: Supplementary file 3 [file mmc3.zip › u022/skilled forgery/Images/u022_s1_f03_Im.png]

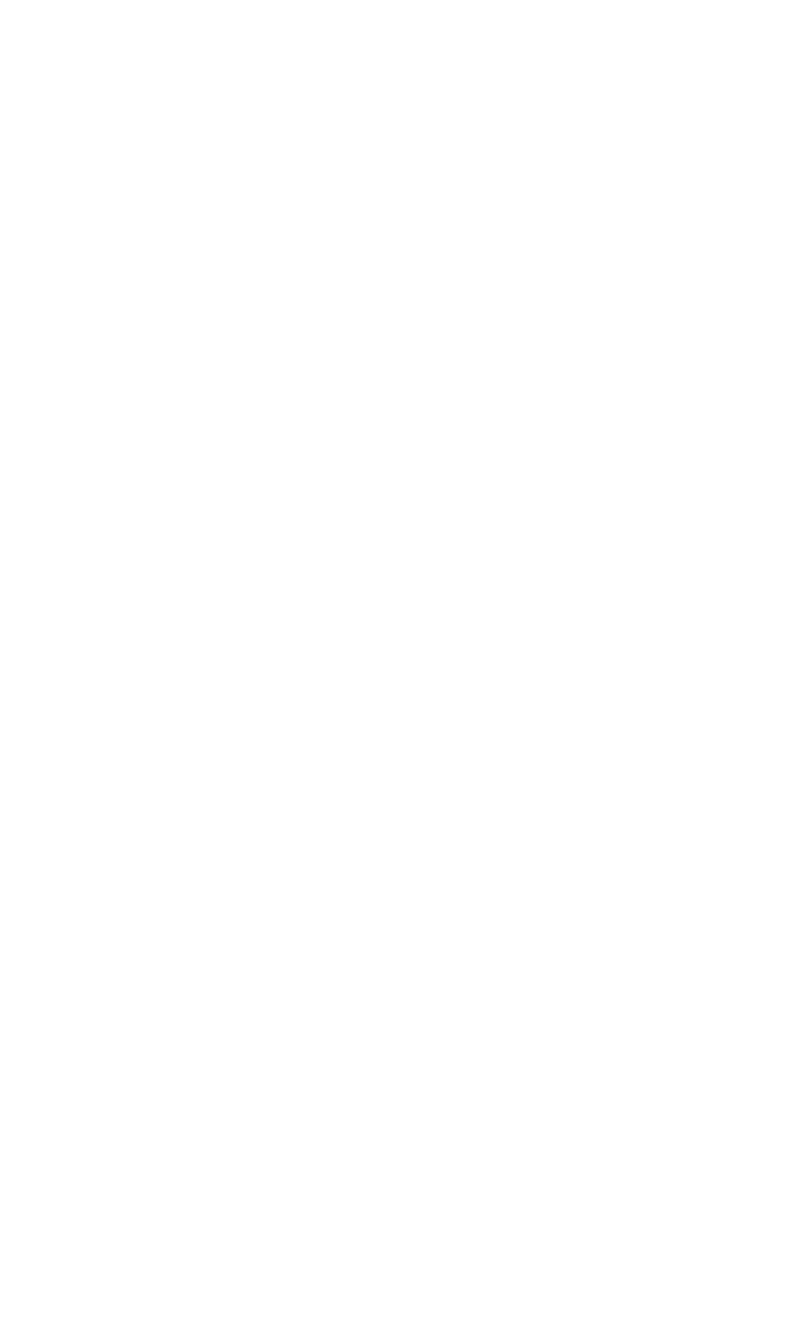

Supplement: Supplementary file 3 [file mmc3.zip › u022/skilled forgery/Images/u022_s1_f04_Im.png]

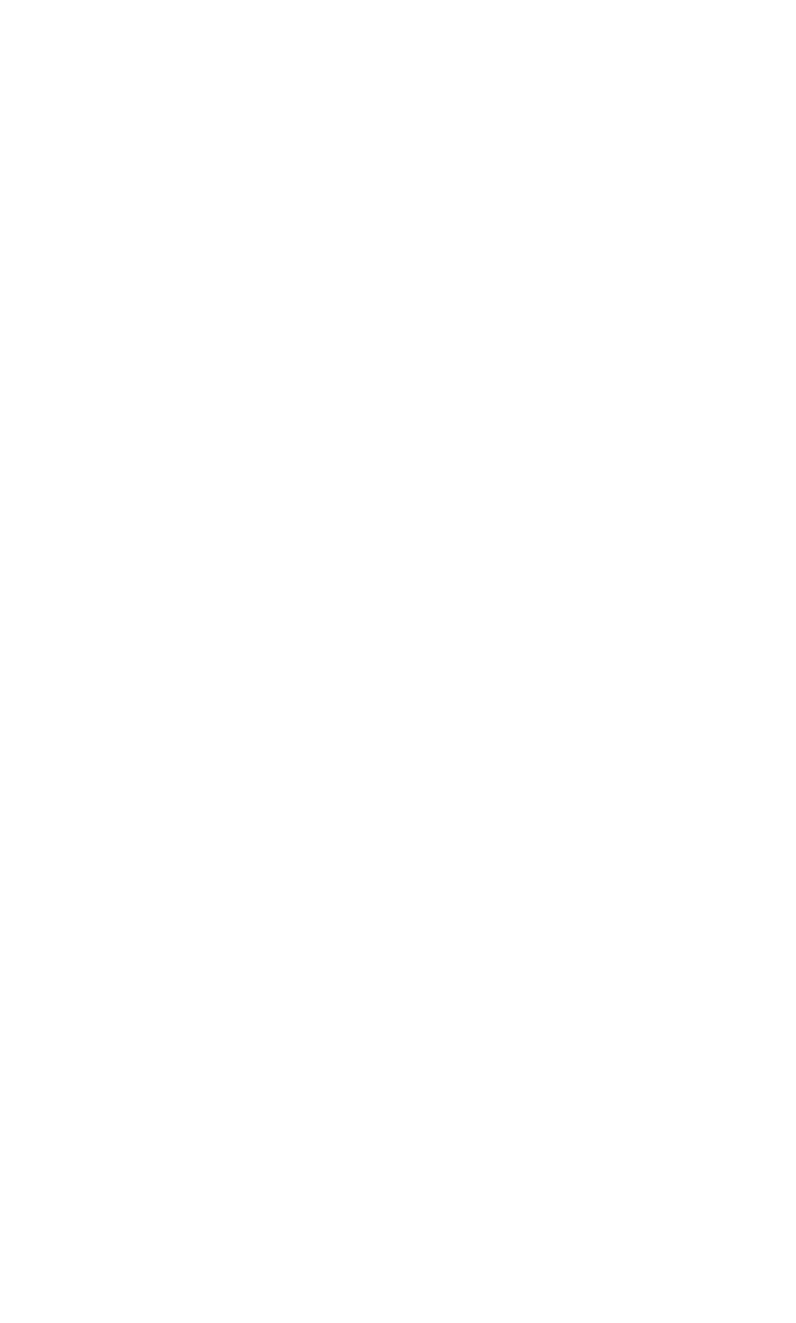

Supplement: Supplementary file 3 [file mmc3.zip › u022/skilled forgery/Images/u022_s1_f05_Im.png]

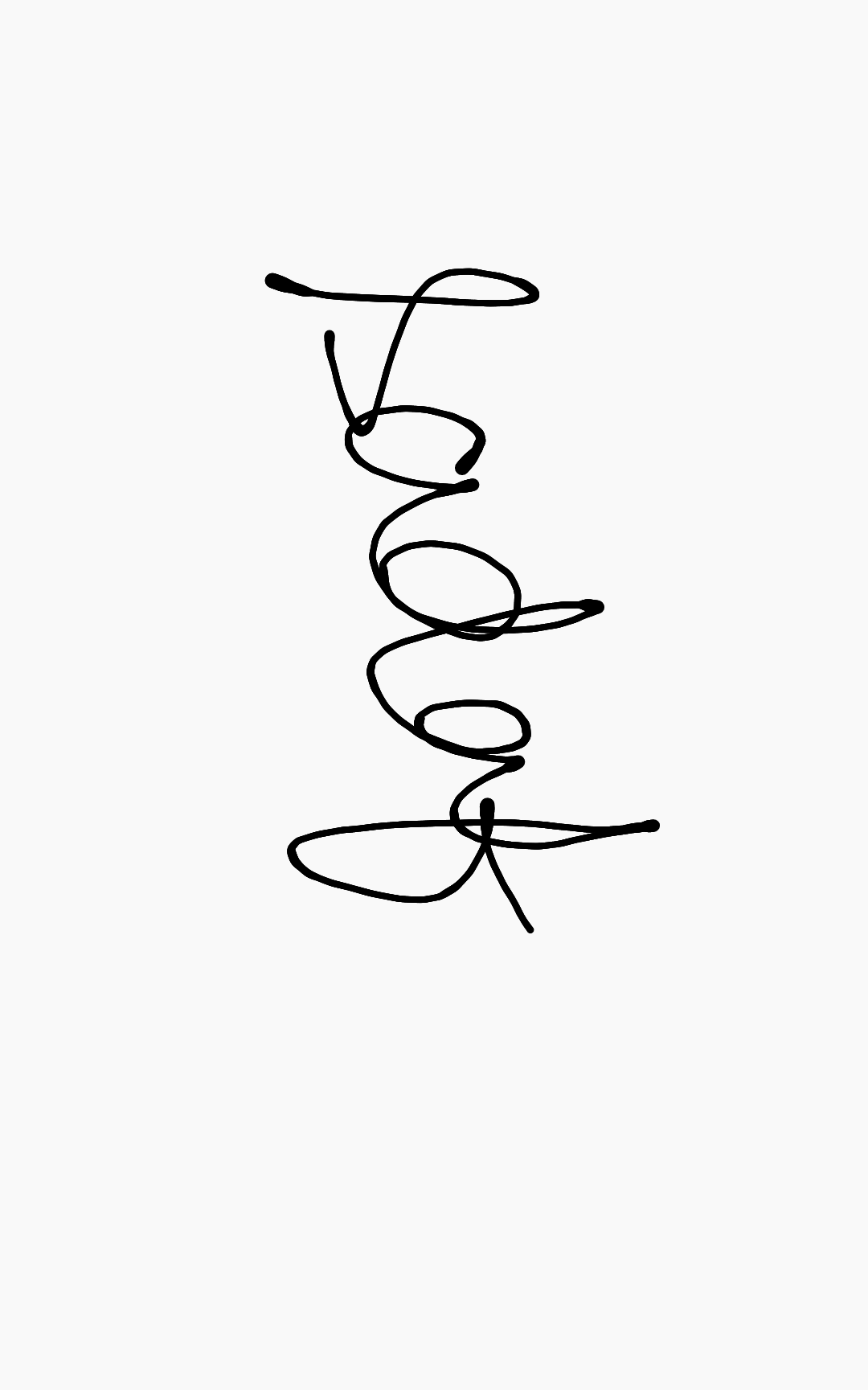

Supplement: Supplementary file 3 [file mmc3.zip › u022/skilled forgery/Images/u022_s2_f01_Im.png]

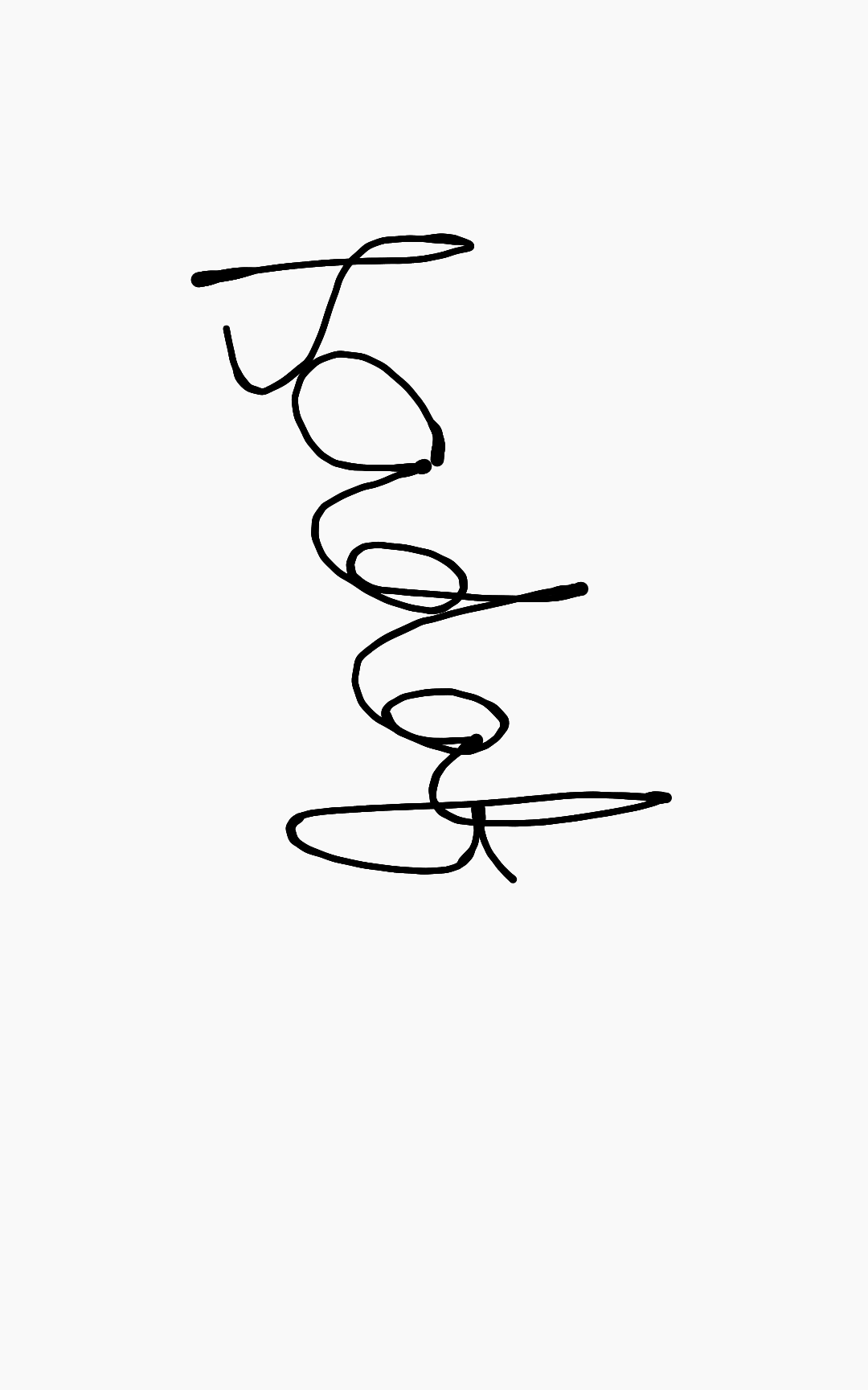

Supplement: Supplementary file 3 [file mmc3.zip › u022/skilled forgery/Images/u022_s2_f02_Im.png]

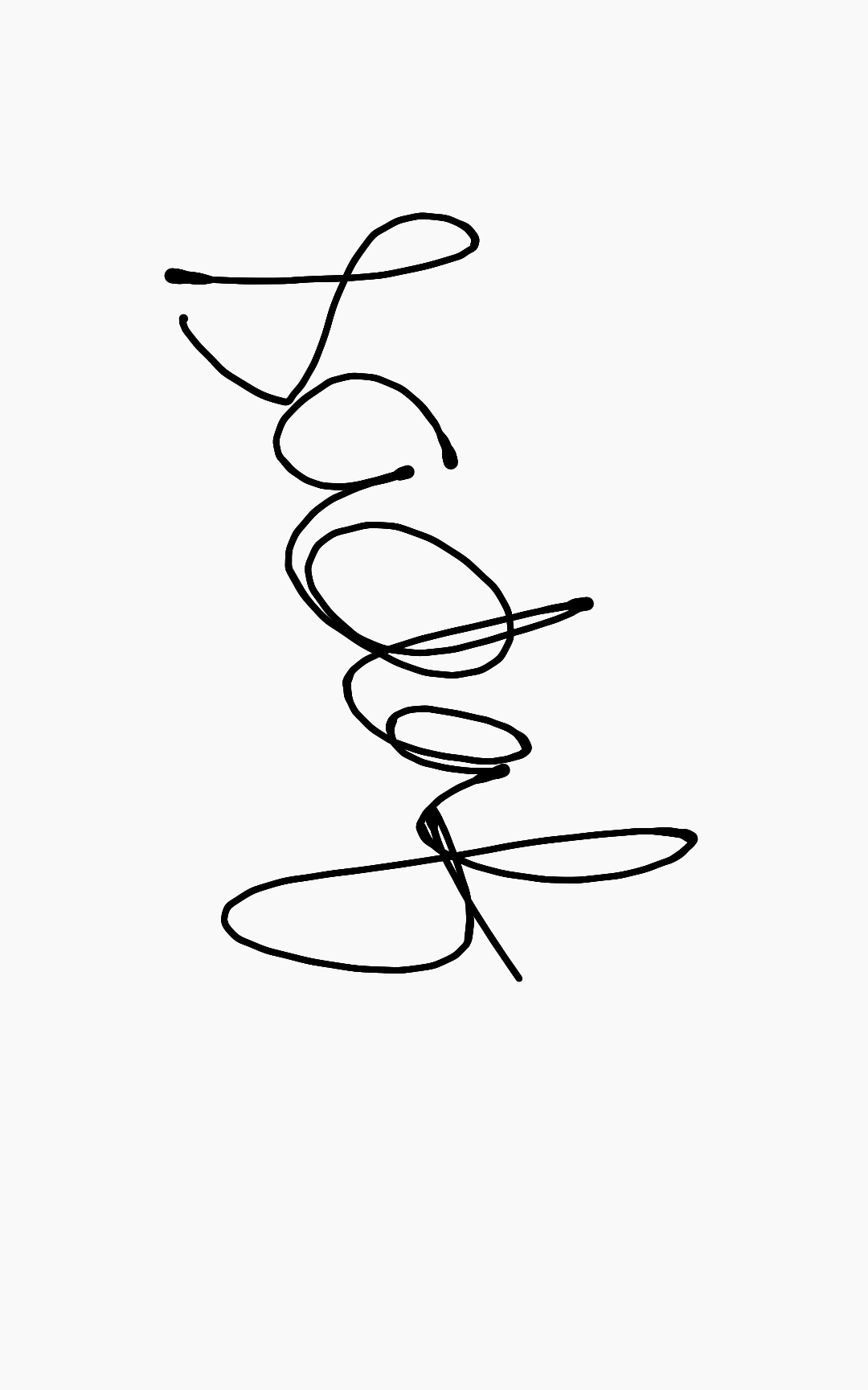

Supplement: Supplementary file 3 [file mmc3.zip › u022/skilled forgery/Images/u022_s2_f03_Im.png]

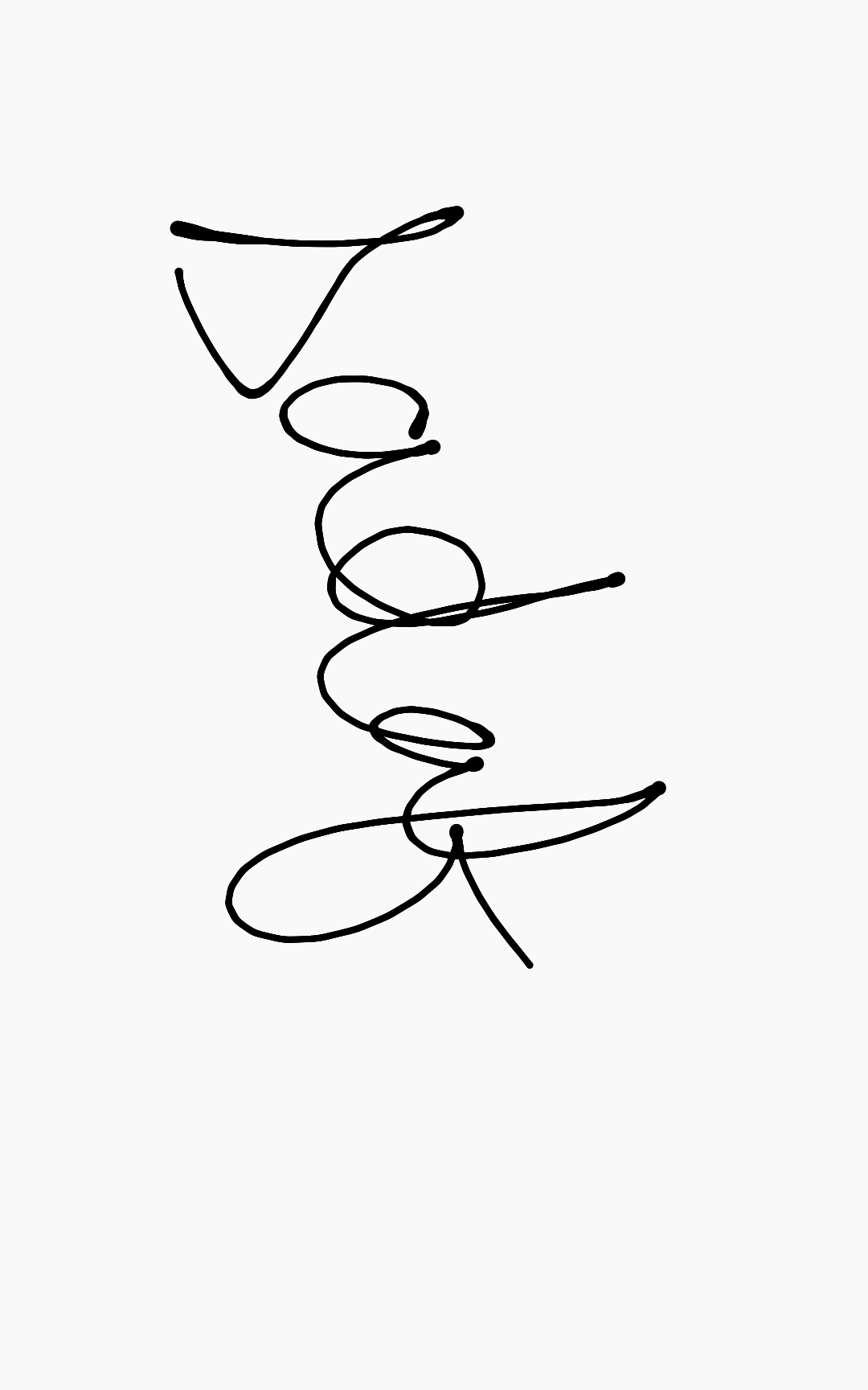

Supplement: Supplementary file 3 [file mmc3.zip › u022/skilled forgery/Images/u022_s2_f04_Im.png]

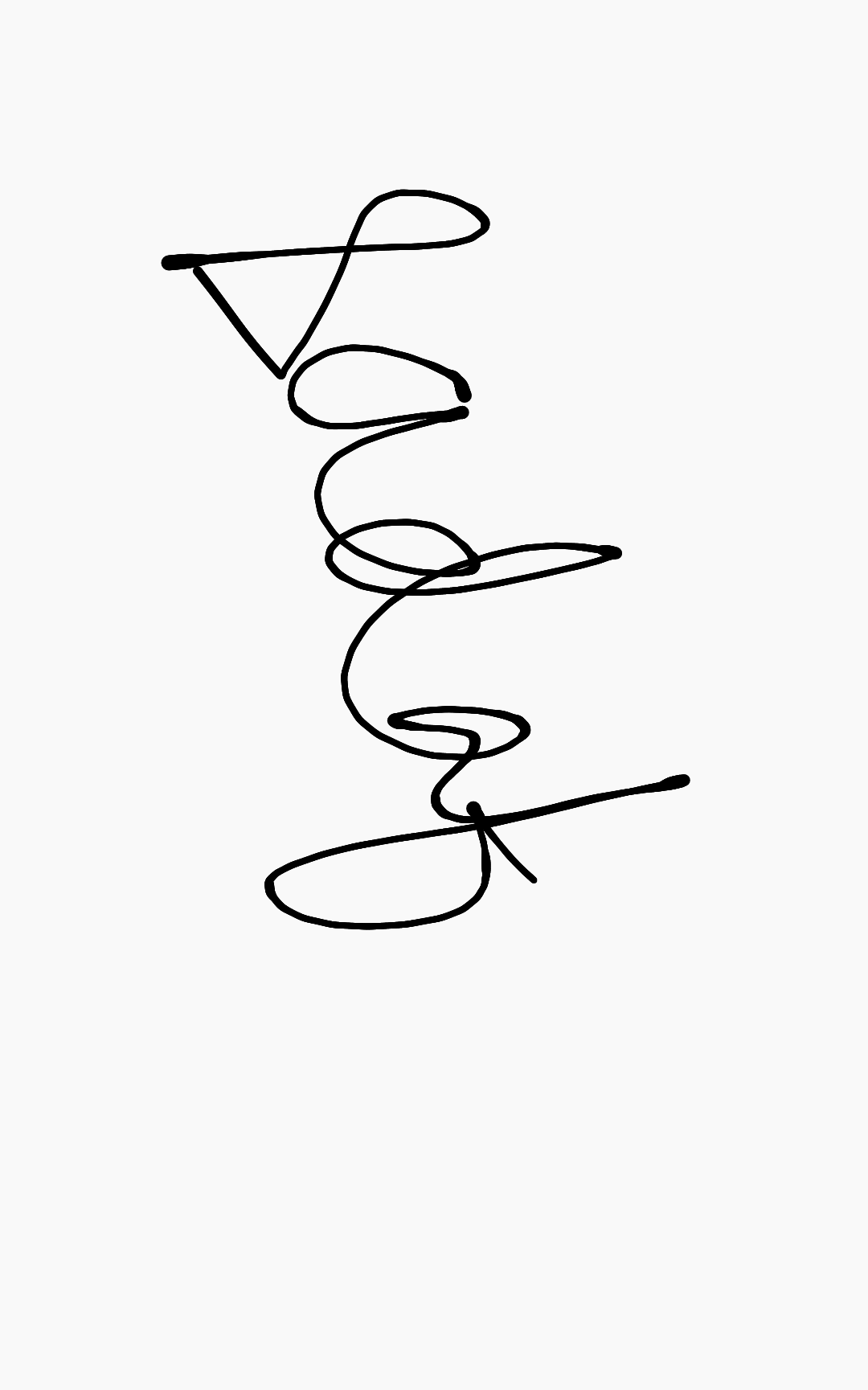

Supplement: Supplementary file 3 [file mmc3.zip › u022/skilled forgery/Images/u022_s2_f05_Im.png]

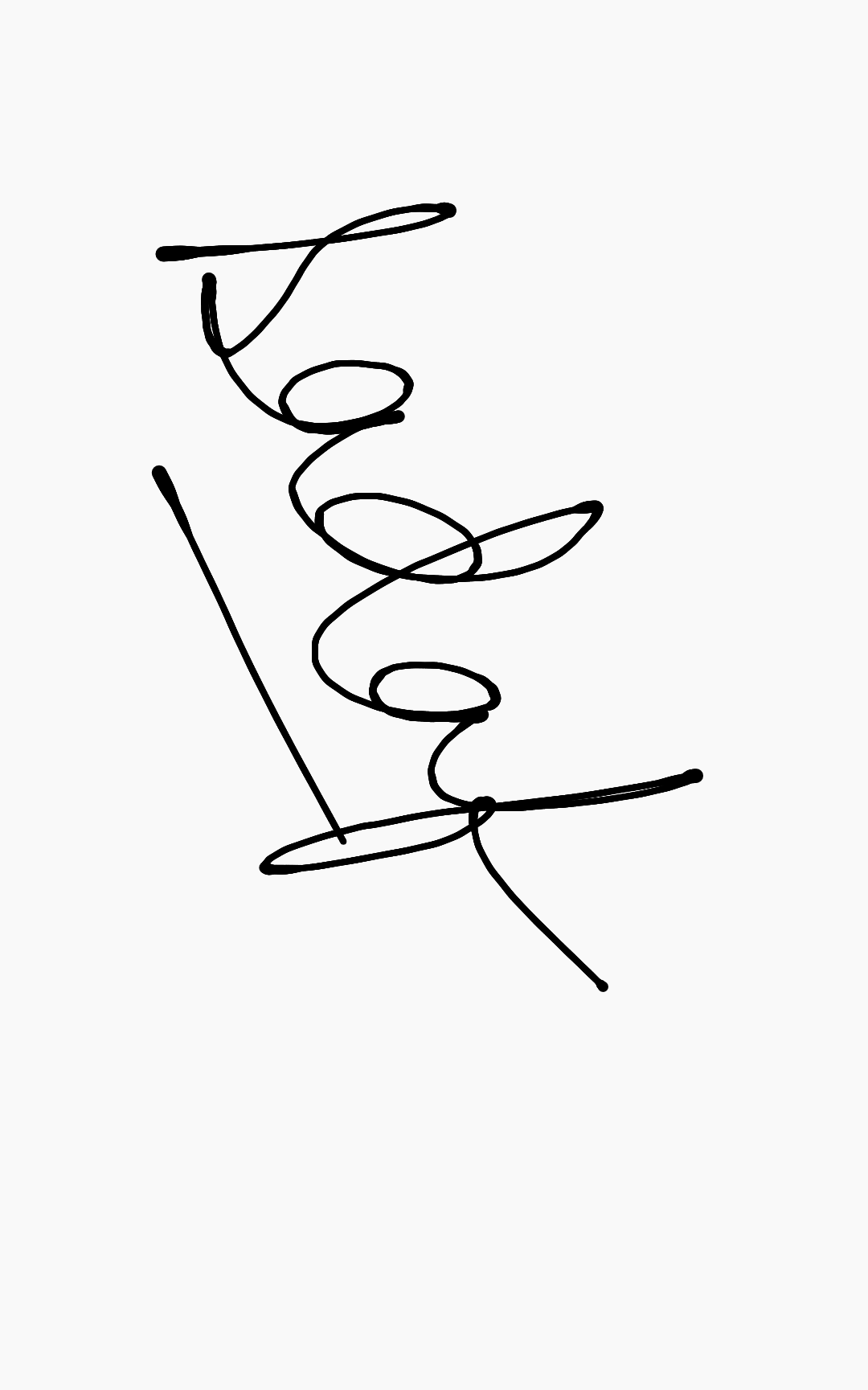

Supplement: Supplementary file 3 [file mmc3.zip › u022/skilled forgery/Images/u022_s3_f01_Im.png]

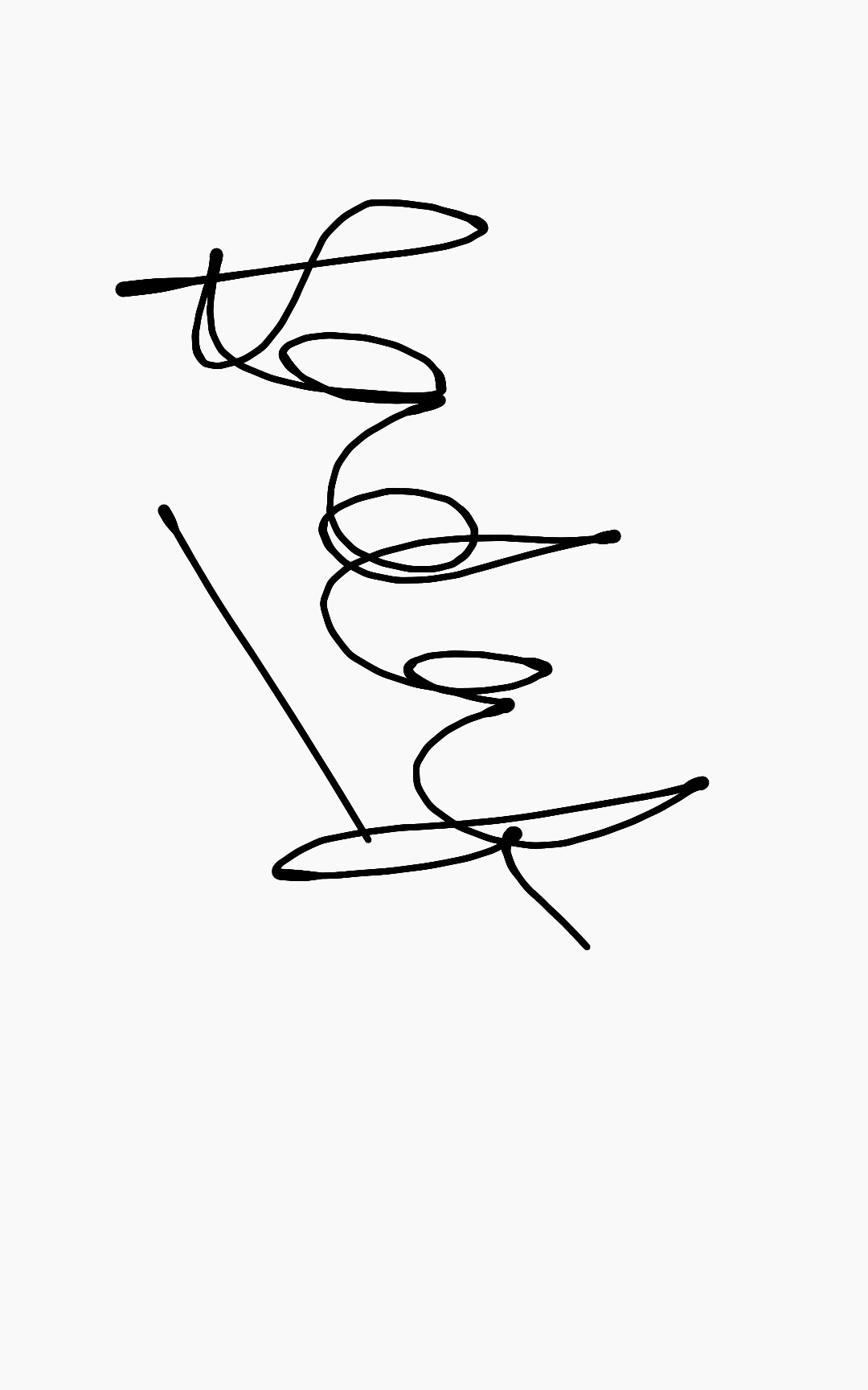

Supplement: Supplementary file 3 [file mmc3.zip › u022/skilled forgery/Images/u022_s3_f02_Im.png]

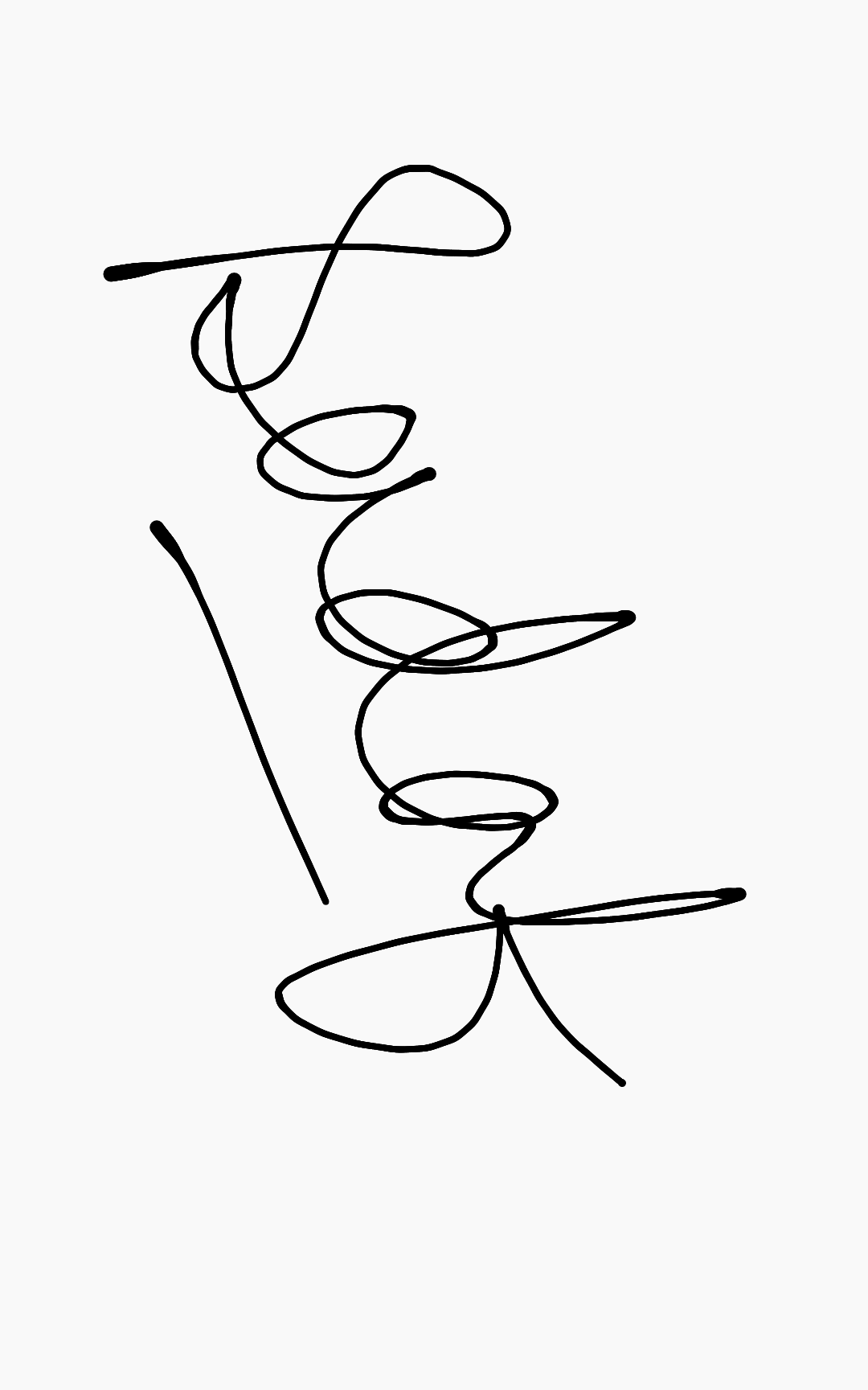

Supplement: Supplementary file 3 [file mmc3.zip › u022/skilled forgery/Images/u022_s3_f03_Im.png]

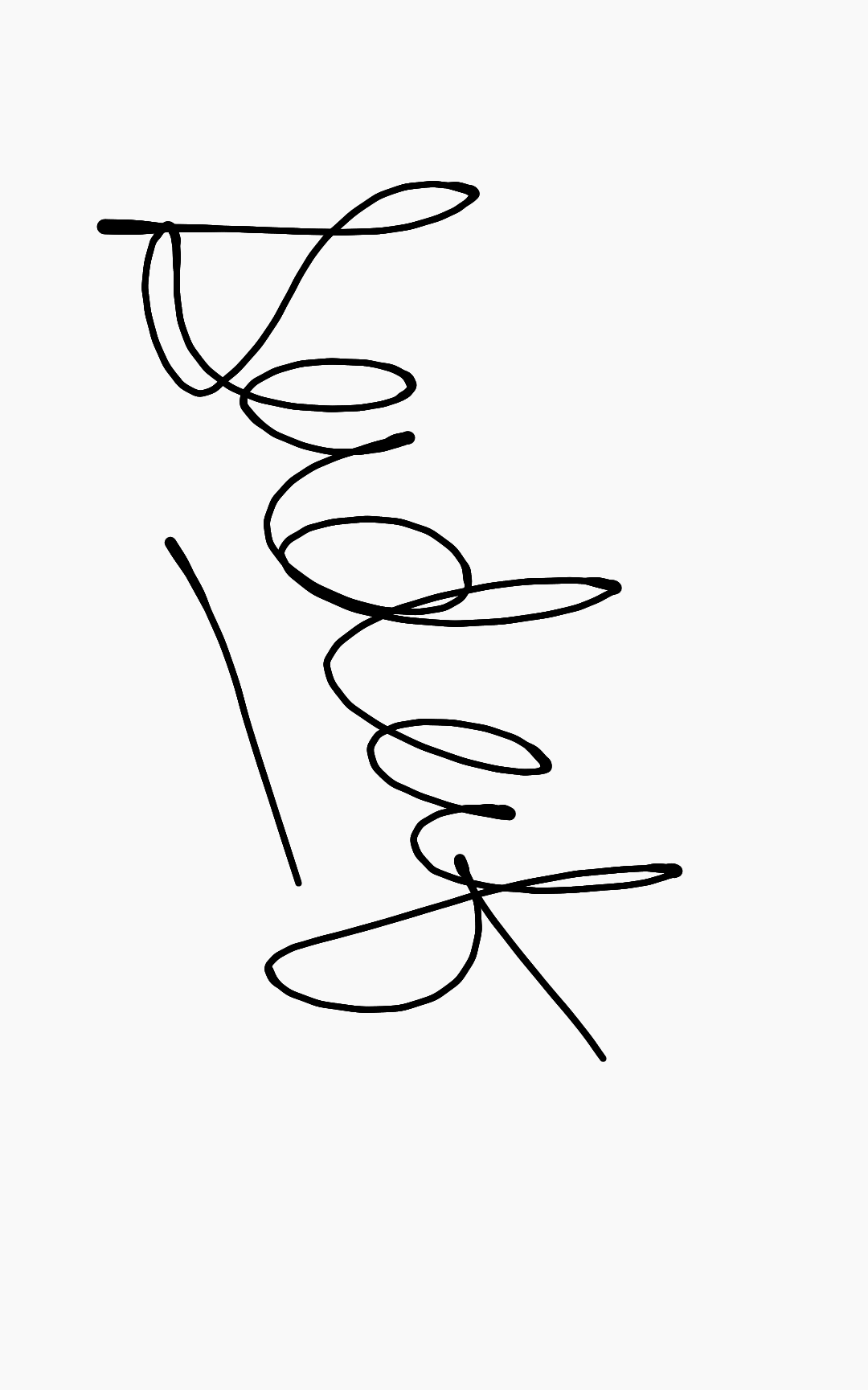

Supplement: Supplementary file 3 [file mmc3.zip › u022/skilled forgery/Images/u022_s3_f04_Im.png]

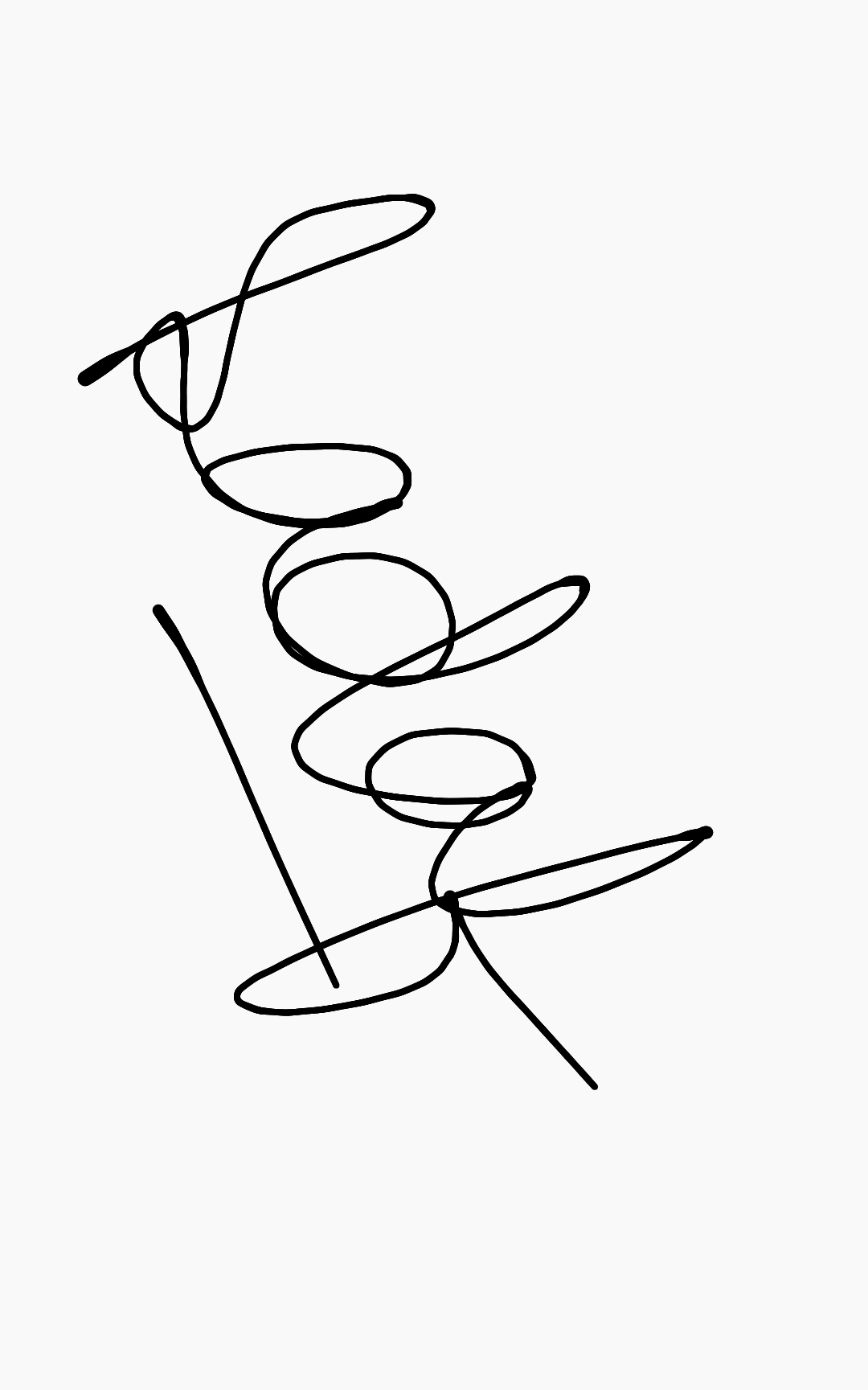

Supplement: Supplementary file 3 [file mmc3.zip › u022/skilled forgery/Images/u022_s3_f05_Im.png]
